# Lymphoid clonal hematopoiesis: implications for malignancy, immunity, and treatment

**DOI:** 10.1038/s41408-022-00773-8

**Published:** 2023-01-04

**Authors:** Kelly von Beck, Troy von Beck, P. Brent Ferrell, Alexander G. Bick, Ashwin Kishtagari

**Affiliations:** 1 Division of Hematology and Oncology, Department of Medicine, Vanderbilt University School of Medicine, Nashville, TN USA; 2 Program in Immunology and Molecular Pathogenesis, Department of Microbiology and Immunology, Emory University, Atlanta, GA USA; 3 Division of Genetic Medicine, Department of Medicine, Vanderbilt University School of Medicine, Nashville, TN USA

**Keywords:** Cancer genetics, Haematological cancer

## Abstract

Clonal hematopoiesis (CH) is the age-related expansion of hematopoietic stem cell clones caused by the acquisition of somatic point mutations or mosaic chromosomal alterations (mCAs). Clonal hematopoiesis caused by somatic mutations has primarily been associated with increased risk of myeloid malignancies, while mCAs have been associated with increased risk of lymphoid malignancies. A recent study by Niroula et al. challenged this paradigm by finding a distinct subset of somatic mutations and mCAs that are associated with increased risk of lymphoid malignancy. CH driven by these mutations is termed lymphoid clonal hematopoiesis (L-CH). Unlike myeloid clonal hematopoiesis (M-CH), L-CH has the potential to originate at both stem cells and partially or fully differentiated progeny stages of maturation. In this review, we explore the definition of L-CH in the context of lymphocyte maturation and lymphoid malignancy precursor disorders, the evidence for L-CH in late-onset autoimmunity and immunodeficiency, and the development of therapy-related L-CH following chemotherapy or hematopoietic stem cell transplantation.

## Introduction

Hematopoietic stem cells (HSCs) are self-renewing, multipotent stem cells that are present in the bone marrow. The total number of HSCs in adult humans are estimated to be in the range of 50,000–200,000 [1]. They renew at a rate of 10^10^-10^12^ cells per day [1] and produce precursors that can develop into all cell types found in peripheral blood. Occasionally, HSCs will acquire a mutation or alteration that confers a competitive advantage by increasing the rate of HSC self-renewal or increasing the apoptotic resistance of subsequent progeny [2]. This eventually creates a genetically identical clone of blood cells that occupies a disproportionate percentage of total blood, a process known as clonal hematopoiesis (CH).

CH is mostly attributed to the gradual selection of mutations in HSCs during aging but can also be induced by external pressures on the HSC niche. Autoimmune destruction of the bone marrow in aplastic anemia creates an evolutionary bottleneck that is associated with CH in up to 50% of cases [3]. Similarly, patients who have received ionizing radiation or chemotherapy in the treatment of solid cancers have a significantly higher rate of CH, ostensibly related or secondary to selection and genomic toxicity provided by the treatment [4].

The first evidence of CH in healthy individuals came from a study in 1994 where 1 in 5 healthy women were found to have skewed X-chromosome inactivation (XCI) within peripheral blood leukocytes, a finding correlated with aging [5]. Further investigation into the cause of XCI skewing culminated in the discovery of somatic mutations in *TET2*, a known driver of hematologic malignancy [6]. This initial discovery paved the way for further investigation in larger datasets. Three groups subsequently performed genome-wide analyses on DNA extracted from the peripheral blood of adults without known hematologic malignancy and found recurrent somatic mutations in 19 known myeloid/lymphoid mutational drivers [7–9]. Steensma et al. proposed the term clonal hematopoiesis of indeterminant potential (CHIP) to describe these otherwise healthy patients with somatic mutations in a known driver of hematologic malignancy at a variant allele frequency (VAF) of ≥2% [10]. CHIP was subsequently found at a rate of 10% in people over the age of 70 and has been associated with an increased risk for cardiovascular disease, hematologic malignancy, stroke, and all-cause mortality [9].

In addition to mutations affecting small segments of DNA, such as point mutations and indels, larger structural variations can also confer a competitive advantage in HSCs [11]. An error in chromosomal replication, such as a gain or loss of chromosomes or a balanced gain-loss event resulting in copy-neutral loss of heterozygosity (cnLOH), is transmitted to the resulting daughter cells, creating a distinct clone of cells with an altered karyotype within an individual [12]. These mosaic chromosomal alterations are commonly observed in both myeloid and lymphoid malignancies and were only recently found to occur in healthy individuals with aging. Large-scale studies demonstrate that the presence of mCAs increases the risk of hematologic malignancy 10-fold [11, 13, 14].

Although both CHIP and mCAs are known risk factors for hematologic malignancy, CHIP had primarily been associated with myeloid malignancies [15], while mCAs have primarily been associated with lymphoid malignancies [11]. While the three 2014 CHIP studies looked at known driver mutations in both myeloid and lymphoid driver mutations [7–9], more recent studies have restricted sequencing to myeloid-associated mutations [16]. Niroula et al. have argued against this notion in their recent elegant study by analyzing CHIP in 55,385 healthy individuals and subclassifying CHIP mutations into driver mutations associated with lymphoid malignancies, termed lymphoid CHIP (L-CHIP), and driver mutations associated with myeloid malignancies, termed myeloid CHIP (M-CHIP) [17]. Similar to CHIP, a distinct subset of mCAs, termed L-mCAs, were associated with lymphoid malignancies, while a separate subset of mCAs, termed M-mCAs, were associated with myeloid malignancies with surprisingly little overlap [11, 13, 14].

**Fig. 1:**
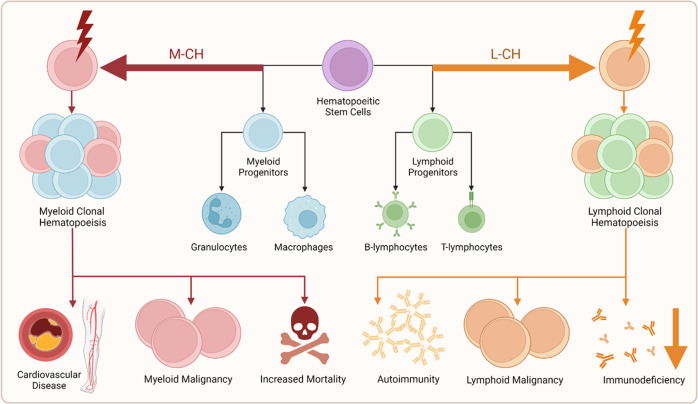
M-CH and L-CH biased mutations drive distinct health outcomes. Mutations acquired in hematopoietic stem cells can provide a selective advantage, leading to the formation and expansion of an HSC clone. Often these mutations bias the production of progeny cells towards either myeloid or lymphoid lineages of cells and have distinct consequences for each lineage. In myeloid biased clonal hematopoiesis (**left**), somatic mutations are associated with an increased risk of myeloid malignancy, cardiovascular disease, and all-cause mortality. In lymphoid biased clonal hematopoiesis (**right**), somatic mutations are associated with lymphoid malignancy and late-onset forms of autoimmunity and immune deficiency.

In the following sections we discuss the evidence for L-CH due to L-CHIP or L-mCAs as a distinct disorder that predisposes patients to a variety of lymphoid malignancies and immune complications (Fig. 1). Table 1 summarizes known key differences between L-CH and M-CH.

**Table 1:** Comparison of Significant Differences between L-CH and M-CH.

	Lymphoid Clonal Hematopoiesis	Myeloid Clonal Hematopoiesis
Frequency of CHIP in Adults aged 40 to 70	1.3% [17]	5.8% [17]
Frequency of mCAs in Adults aged 40 to 70	0.8% [17]^a^	0.4% [17]^a^
Most Frequent CHIP Mutations and Distribution	DUSP22, FAT1, KMT2D, SYNE1, and ATM mutations account for ~20% of L-CHIP variants [17]	DNMT3A, TET2, and ASXL1 mutations account for 87% of M-CHIP variants [17]
Most Frequent mCAs	Tri12, LOH_ITPKB, Del13q, LOH_MIR16-1, LOH_NOTCH1, Del10q [17]^b^	LOH_TCL1A, Del20q, LOH_EP300, LOH_JAK2, Del5q, Tri8 [17]^b^
Stages of maturation where mutations may occur	HSCs, immature lymphoid precursors, and mature lymphocytes	HSCs and immature myeloid precursors

^a^Note that 65% of mCAs were not classified as L-mCA or M-mCA in this cohort leading to a potential underestimation of frequency.

^b^LOH, Loss of Heterozygosity; Del, Deletion, Tri, Trisomy.

## Definition of L-CH in the context of lymphocyte maturation

Compared to bone marrow-derived myeloid cells, lymphocytes of the B and T lineages possess a complex differentiation pathway and are unique in their capacity to persist for the life of the host and expand following stimulation [18–22]. These characteristics call for an additional level of nuance when describing CH in lymphoid populations, with particular attention paid to whether driver mutations are acquired in a bone marrow progenitor or in differentiated cells in the periphery.

**Fig. 2:**
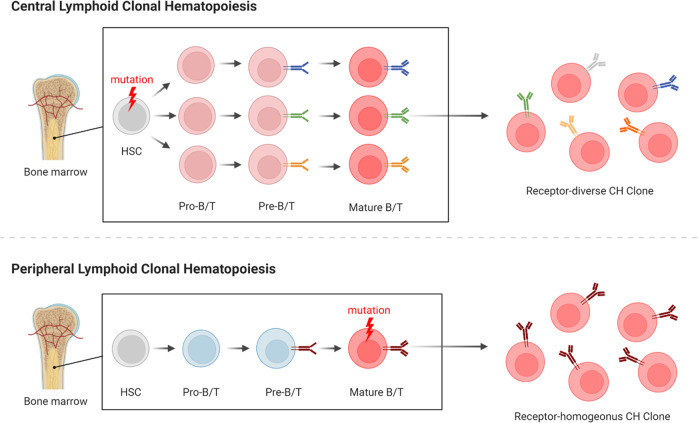
Lymphocyte Receptor Diversity in Clonal Hematopoiesis. During lymphocyte development, HSC’s follow a multi-step differentiation pathway which includes the rearrangement of BCR or TCR genes for B cells and T cells respectively. Receptor rearrangement defines distinct stages of lymphocyte development with the rearrangement of the immunoglobulin heavy chain or TCRβ defining the transition from pro-B/T to pre-B/T cells and the subsequent rearrangement of the immunoglobulin light chain or TCRα chain occurring during the transition from pre-B/T to mature B/T cells. Stochastic rearrangement of lymphocyte receptor genes in each developing cell produces a diverse repertoire of B and T cells. (**Upper**) CH mutations which occur in the HSC, defined here as central lymphoid clonal hematopoiesis, are carried forth in all progeny cells which then undergo receptor rearrangement to form a receptor-diverse CH clone. (**Lower**) CH mutations occurring in mature lymphocytes which have already completed receptor rearrangement, defined here as peripheral lymphoid clonal hematopoiesis, will expand and form a receptor-homogenous CH clone.

Mature myeloid cells are notably short-lived and lacking in proliferative potential, thus, mutations typically occur prior to or early on in myeloid commitment, such as the HSC compartment. This HSC mutation origin also applies for L-CH candidate genes in lymphocytes; however, the long-term persistence of mature lymphocytes coupled with their proliferative capacity and programmed mutagenesis following cognate antigen exposure creates additional opportunities where mature cells can acquire L-CH mutations. Mutations in precursors and mature lymphocytes will both produce a clonal population; however, mutations occurring before receptor rearrangement in the HSC compartment will produce B and T-cells with unique receptors, while those affecting mature cells in the periphery will generate a population of either B or T-cells with an identical lymphocyte receptor (Fig. 2) [23]. We denote these distinct entities as either “central” or “peripheral” L-CH depending on whether the mutated cell has completed rearranging its lymphocyte receptor. Although central L-CH produces an antigen receptor diverse population of naïve lymphocytes, malignancy may still appear monoclonal if only a single lymphocyte from the CHIP clone expands to cause disease [23]. The central and peripheral forms of L-CH pose unique challenges for treatment and distinct risk profiles for lymphoma, autoimmunity, and immune deficiency, which are discussed in greater detail in following sections. Finally, excluding pro/pre-lymphocyte malignancies, the distinction of central L-CH from peripheral L-CH can only be made when studies either directly sequence the progenitor compartment, sequence multiple lineages in isolation, or use single cell sequencing techniques to identify receptor-diverse L-CH clones. We are therefore unable to infer the contribution of central and peripheral L-CH in each disease setting but include the data as available.

## L-CH in large-cohort clonal hematopoiesis studies

Multiple large-scale cohort studies have examined both myeloid and lymphoid malignancy driver mutations in individuals without a known hematologic disorder (summarized in Table 2), and a growing list of L-CHIP mutations have been incidentally identified. Jaiswal et al. analyzed DNA from peripheral blood cells of 17,182 persons who were unselected for hematologic phenotypes and discovered 45 pathogenic variants in 28 genes associated with lymphoid malignancy. Although the associated mutations are reported for several patients with post-CHIP hematologic malignancy, the low overall burden of hematologic malignancy in this cohort precluded any distinction of L-CHIP or M-CHIP [9]. Zink et al. performed whole genome sequencing in 11,262 Icelanders and found 17 pathogenic variants in 8 genes associated with lymphoid malignancy [24]. However, they did not distinguish between myeloid and lymphoid neoplasms or differentiate between myeloid and lymphoid malignancy-associated mutations in their analyses. Both studies found that CHIP mutations significantly increase the risk of hematologic malignancy. One seminal study investigated CHIP mutations at a much lower VAF threshold (0.0003) than was previously published and found four L-CHIP mutations in 3 patients that were present in all hematopoietic lineages, suggesting HSC origin [25].

**Table 2:** Summary of major studies associating L-CH with lymphoid malignancy.

Publication	Technique	Cohort	Major Findings	Ref.
Jacobs et al. (2012)	Detection of mCAs in blood from one or more Infinium Human SNP Arrays	57,853 individuals from 13 genome-wide association studies	22% of individuals with lymphocytic leukemia had detectable clonal mosaicism at least 1 year prior, compared to 0.74% of cancer-free individuals. CLL was the predominant variety of lymphocytic leukemia, with frequent mosaic deletion of 13q14.	[13]
Laurie et al. (2012)	Detection of mCAs in blood from one of five Illumina array types	8,562 individuals from 15 studies belonging to the Gene Environment Association Studies consortium	Of 15 individuals with clonal mosaicism and subsequent hematologic cancer, 6 would develop CLL and 1 would develop MM. Of the 6 CLL cases, 5 bore the 13q deletion.	[14]
Schick et al. (2013)	Detection of mCAs in blood from one of four Illumina array types	12,176 individuals from the Group Health electronic Medical Records and Genomics study and the Women’s Health Initiative	mCAs appear to rarely precede the development of MM (1/46) or HL (0/6) by greater than 1 year.	[26]
Barrio et al. (2017)	Detection of somatic mutations by targeted deep sequencing of 24 genes implicated in CLL	48 individuals with high-count MBL	Mutations in high-count MBL significantly overlap with CLL. MBL carrying a mutation in one of 24 CLL genes posed a greater risk to CLL progression than MBL with no somatic mutation detected. Subclonal expansion of mutation-carrying MBL clones is predictive of progression to CLL.	[37]
Agathangelidis et al. (2018)	Detection of somatic mutations using Illumina Nextera WGS kit (VAF > 10%)	29 individuals with either MBL or CLL diagnosis	Somatic mutations identified in MBL significantly overlap with CLL driver genes.	[36]
Loh et al. (2018)	Detection of mCAs in blood from Affymetrix UK BiLEVE and UK Biobank AXIOM microarrays	152,248 individuals from the UK Biobank between 40 and 70 years of age at time of blood collection.	Incidence of CLL is positively correlated with the presence of Trisomy 3 and 12 as well as 13q LOH. Size of the clonal fraction is inversely correlated with the time to CLL diagnosis.	[11]
Terao et al. (2020)	Detection of mCAs in blood from one or more Infinium Human SNP Arrays	179,417 individuals from the Biobank Japan cohort	Common mutational precursors of CLL (trisomy 12, 13q deletion, and 13q LOH) were less frequent in Japanese individuals compared to the UK Biobank cohort. Selective pressure for specific mCAs varies with genetic background, consistent with the lower rates of CLL and higher rates of T-cell leukemia in the Japanese.	[28]
Niroula et al. (2021)	Detection of SNVs/indels in WES data at 56 M-CH related genes and 235 genes recurrently mutated in lymphoid malignancy (VAF > 2%). mCAs were previously detected in a prior study. CH and mCAs were excluded if they were detected less than 6 months before a hematologic malignancy diagnosis.	420,969 individuals with SNP microarray data and 55,383 individuals with WES from the UK Biobank and Mass General Brigham Biobank	CHIP occurs at both M-CHIP/M-mCA and L-CHIP/L-mCA loci and increases with age. M-CH and L-CH loci clearly segregate on the incidence myeloid and lymphoid malignancy. M-CHIP is dominated by DNMT3A, TET2, and ASXL1 somatic mutants, while L-CHIP is spread more evenly across a large number of genes. CLL was the dominant lymphoid malignancy occurring subsequent to L-CH.	[17]
Saiki et al. (2021)	Detection of SNVs/indels by targeted sequencing of 23 CH-associated genes (predominantly M-CH, VAF > 0.5%) and detection of CNAs by one of three Illumina array types	11,234 individuals without hematologic malignancy from the Biobank Japan cohort	Detection of CH strongly correlated with subsequent death attributable to myeloid malignancy (160/215) but only weakly with lymphoid malignancy (191/420) compared to non-hematologic malignancy death controls (4,209/10,562). These observations held true for all myeloid malignancy subtypes and lymphoid malignancy subtypes except CLL (7/7) and ALL (7/12).	[27]
Schroers-Martin et al. (2021)	Detection of somatic mutations by targeted deep sequencing of commonly mutated lymphoma driver genes.	29 individuals with subsequent follicular lymphoma diagnosis and 34 healthy controls from the American Cancer Society Cancer Prevention Study-II LifeLink cohort	Somatic mutations in t(14;18) positive follicular lymphoma can be detected years prior to diagnosis in low-frequency t(14;18) positive precursor cells.	[46]
Coffey et al. (2021)	Detection of somatic mutations in immunophenotyped blood cells by targeted single-cell DNA sequencing of 22 genes recurrently mutated in MM and CHIP	12 individuals with diagnosed MM	Somatic mutations detected in peripheral blood plasma cells were also identified in other hematopoietic lineages, indicating a bone marrow mutational origin for the MM precursor lesion.	[59]
Kar et al. (2022)	Detection of SNVs/indels by WES of peripheral blood DNA with the IDT xGen Exome Research Panel v1.0. SNVs/indels were characterized for 43 CH-related genes (predominantly M-CH)	200,453 individuals from the UK Biobank	Among the 43 CH-related genes, the incidence of lymphoid leukemia only increases significantly when the VAF exceeded 10% without an incident myeloid malignancy	[16]

In their seminal paper, Niroula et al. differentiated M-CHIP and L-CHIP using the UK Biobank and Mass General Brigham Biobank [17]. They analyzed whole exome sequencing (WES) data from 46,706 individuals without hematologic malignancy and found 617 variants in 234 genes associated with lymphoid malignancy. Like M-CHIP, the prevalence of L-CHIP increased with age. It was less common than M-CHIP in the study population (1.3% vs. 5.8%) with variants that were more evenly distributed across a larger number of genes. L-CHIP was found to be associated with increased risk of chronic lymphocytic leukemia (CLL)/small lymphocytic lymphoma (SLL) and was not associated with an increased risk of myeloid malignancy. Similarly, M-CHIP was associated with an increased risk of myeloid malignancy but not lymphoid malignancy. It should be noted that while individual genes may bias towards myeloid or lymphoid malignancy, they were not exclusive. Many of the most frequently mutated CHIP genes, such as *DNMT3A* and *TET2* coincided with cases of both lymphoid and myeloid malignancies. Although CHIP has previously been associated with increased risk of coronary artery disease and all-cause mortality, L-CHIP was not associated with either outcome in this study [17].

Similarly, multiple large-scale studies have examined DNA from peripheral blood for mCAs in healthy individuals (summarized in Table 2), and 125 classes of mCAs have been identified to date, ~75% of which are primarily associated with lymphoid malignancy [11, 13, 14, 26, 27]. The frequency of specific mCAs is further influenced by the genetic background of the individual [28]. Overall, mCAs due to copy number gain or loss are more frequent in men (OR = 1.42) [13], while cnLOH is distributed equally among the sexes. The prevalence of mCAs is low (<0.5%) in individuals under 50 but increases to 2-3% in the elderly population. These studies found that mCAs are associated with a greater than 10-fold increased risk of any hematologic cancer as well as an increased risk of CLL [14]. Niroula et. al was the first to differentiate between L-mCA and M-mCA [17]. They found that L-mCAs were associated with increased risk of CLL, diffuse large B-cell lymphoma, follicular lymphoma, and overall non-Hodgkin lymphoma (NHL), while M-mCAs were almost exclusively associated with myeloid malignancies.

## L-CH and lymphoid malignancy precursor disorders

### Monoclonal B-cell lymphocytosis

The term monoclonal B-cell lymphocytosis was first coined by Marti et al. [29]. after multiple studies found circulating populations of monoclonal B-cells in 2-3% of otherwise healthy individuals [30, 31]. It was further defined as the detection of a monoclonal population of B-cells <5×10^9^/L in peripheral blood with the absence of lymphadenopathy, organomegaly, or any other feature diagnostic of a B-lymphoproliferative disorder [29]. The disorder was further subcategorized into low- and high-count MBL after a population screening study in 2010 demonstrated a distinctly bimodal distribution of monoclonal B-cell counts, with most patients exhibiting counts between 0.1 and 0.5×10^9^/L and higher-risk patients exhibiting counts between 0.5 and 5 ×10^9^/L with strikingly little overlap [32]. High-count MBL progresses to CLL at a rate of 1-5% per year [33] while low-count MBL has not been associated with progression [34]. Three distinct subtypes based on immunotypic features have been identified: CLL-like MBL (CD19^+^, CD20^dim^, CD5^+^, CD20^+^, surface Ig^dim^, FMC7^-^), atypical CLL-like MBL (CD19^+^, CD20^bright^, CD5^+^, CD23^-^, FMC7^+/-^), and CD5^-^ MBL which resembles marginal zone lymphoma [35]. Up to 75% of cases are CLL-like MBL, while the remaining 25% are split between atypical CLL-like MBL and CD5^-^ MBL. Atypical CLL-like and CD5^-^ cases of MBL tend to be transient, while 90% of CLL-like MBL cases persist over time [34].

In their seminal paper, Niroula et al. found a significant association between L-CHIP and risk of CLL (HR = 20.5) but were unable to assess the relationship between L-CHIP and MBL [17]. However, there is significant overlap between the L-CHIP mutations described by Niroula et al. and the putative lymphoid driver mutations that have previously been reported in patients with CD19^+^/CD5^+^/CD20^dim^ MBL. Agathangelidis et al. identified mutations in 7 CLL driver genes in the MBL cells of 11 patients with CD19^+^/CD5^+^/CD20^dim^ MBL including *NOTCH1*, *FBXW7*, and *POT1* [36], all of which were identified as L-CHIP mutations. Subsequent work found recurrent mutations in 8 CLL driver genes in the MBL cells of 25/48 patients with high-count CD19^+^/CD5^+^/CD20^dim^ MBL [37], which were later identified as L-CHIP. Niroula et al. found that L-mCA is associated with CLL (HR = 68.6) [17] demonstrating greatly increased risk of progression to malignancy compared to the normal population. CD19^+^/CD5^+^/CD20^dim^ MBL is classically known to possess L-mCAs characteristic of CLL, including del 13q, trisomy 12, del 11q, and del 17p in up to 50% of cases [38]. The presence of del 13q or trisomy 12 + del 17p in CD19^+^/CD5^+^/CD20^dim^ MBL are associated with increased risk of progression to CLL [39]. Other types of mCAs have not been investigated in MBL cohorts.

As described above, there is significant overlap between CD19^+^/CD5^+^/CD20^dim^ MBL and L-CHIP. However, the current clinical definition of MBL is more restrictive as it only captures cases of peripheral L-CH. The current diagnosis of MBL is based on flow cytometric detection of kappa to lambda light chain ratio skewing or abnormal surface markers associated with a cancer phenotype [29]. However, if the clonal mutation occurs prior to light chain selection as in the case of central CH, the kappa to lambda ratio will be balanced as normal. In fact, a recent study found detectable IGH skewing in 21/28 patients up to 15 years prior to CLL diagnosis and most commonly in the absence of detectable monoclonal lymphocytosis [40].

While the vast majority of patients with L-CH likely have CLL-like MBL, there may be a significant subset of patients at risk for CLL without a detectable monoclonal lymphocytosis. Patients with an absolute lymphocyte count >5 ×10^9^ should be worked up for neoplastic or reactive causes with a complete history, physical examination, and blood smear [41]. Polyclonal lymphocytosis can be related to viral or bacterial infection, autoimmunity, or stress. Central L-CH should be a potential consideration in patients with persistent unexplained polyclonal lymphocytosis.

### Premalignant lymphoma

Lymphomas are clonal proliferations of lymphocytes that are broadly categorized by lineage (B-cell, T-cell, NK cell) and maturity of the clone. It is now widely recognized that cancer development is a protracted process that is universally preceded by precursor lesions. Unlike MBL and MGUS, which have strict definitions and criteria, the precursor states of lymphomas are less well-defined, and the lack of bone marrow or blood infiltration makes molecular studies more difficult in this context.

A growing body of evidence points towards a stepwise progression in the development of follicular lymphoma, the second most common form of NHL. Follicular lymphoma is characterized by the proliferation of germinal center-like B-cells that aberrantly express *BCL2* via translocation to the IGH region, t(14;18), in >85% of cases [42]. However, B-cells carrying the t(14;18) translocation can be detected at low levels in the peripheral blood of 46% of healthy individuals [43]. Further studies found that the presence of circulating t(14;18)-positive B-cells was significantly associated with the development of follicular lymphoma (OR = 2.8), and that the circulating t(14;18)-positive B-cells were clonally related to the lymphoma B-cells [44]. These findings show that *BCL2* translocations are necessary but not sufficient for lymphomagenesis. BCL2 translocation likely represents the first step in follicular lymphomagenesis given its pervasiveness, but these studies demonstrate that it is a weak oncogene requiring further mutations to complete the transformation to follicular lymphoma.

One interesting case report describes the development of genetically similar follicular lymphomas in both donor and recipient 7 years after hematopoietic stem cell transplant, suggesting that a common follicular lymphoma precursor was present in the bone marrow [45]. The presence of 11/12 lymphoma driver mutations in both follicular lymphomas and a preserved sample from a donor lymphocyte infusion 7 years prior implicates L-CHIP as a potential precursor lesion in the development of follicular lymphoma. Schroers-Martin et al. looked for lymphoma driver mutations in circulating t(14;18)-positive B-cells of patients who would later develop follicular lymphoma and found driver mutations in 24% of cases at a median time to diagnosis of 44 months. The most common driver mutations identified were *CREBBP* (40%) followed by *BCL2* (23%) [46]. The presence of lymphoma driver mutations in these healthy patients who will eventually develop follicular lymphoma illustrates the potential role of L-CHIP in lymphomagenesis.

There is more evidence for the role of L-CHIP in the development of follicular lymphoma compared to other lymphomas, but it is likely a similar model of pathogenesis applies. Other lymphoma-related translocations have been identified in the blood of healthy individuals including t(11;14) [47], the primary translocation implicated in mantle cell lymphoma, and t(2;5) [48], a translocation most commonly associated with diffuse large B-cell lymphoma and Hodgkin lymphoma.

### Monoclonal gammopathy of undetermined significance

Monoclonal gammopathy of undetermined significance (MGUS) is a precursor disorder to multiple myeloma (MM) [49]. MGUS is present in 3.2% of individuals over age 50 and 5.3% of individuals over age 70 [50]. The risk of progression of MGUS to MM is about 1% per year [51]. MGUS and MM are diseases that are localized to the bone marrow, requiring the sorting of often small numbers of plasma cells from bone marrow extract in order to perform molecular analyses. MGUS and MM can be further classified by immunoglobulin subtype with IgG being the most frequent [50]. Although less common, the IgA subtype of MGUS is associated with a higher risk of progression to MM [52].

The germinal center has been traditionally thought of as the primary site for myelomagenesis. After exposure to their target antigen, B-cells migrate to the germinal center and undergo somatic hypermutation of the DNA encoding the immunoglobulin variable regions to produce an antibody with stronger antigen binding, a process termed affinity maturation. The antibodies are further optimized for a targeted immune response through the process of class switch recombination (CSR). During these processes, DNA double-strand breaks are generated, and though most are successfully repaired, some aberrantly translocate to other locations within the genome [53]. This creates translocations and mCAs which are highly characteristic of MM and are found in almost all cases [54]. Translocations and mCAs are not, on their own, sufficient for myelomagenesis as further mutations must be acquired to complete the transformation to MM [55].

Subsequent work has challenged the notion that all translocations and mCAs are acquired during CSR, showing that a significant number are generated during Recombination-activating gene (RAG) mediated recombination at V(D)J joins rather than at CSR switch regions [56]. This appears especially common in the development of IgM-MM, where translocations involving the joints between V, D, and J genes occur in 60% of IgM-MM cases compared to only 25% of all other MM isotypes [57].

Evaluation of the role of L-CHIP in the pathogenesis of MGUS and MM using currently available research is limited because mutational analyses have been predominantly limited to tumor biopsy samples. The exact timing of driver mutation acquisition has not been well-studied for most genes, although *KRAS* mutations have been shown to be secondary events [58]. Coffey et al. showed that some driver mutations found in MM tumor biopsy samples are present across multiple hematopoietic cell lineages, indicating that the mutations occurred in a common ancestor progenitor cell [59]. Based on these findings, we hypothesize that pre-germinal center driver mutations appear in circulating B-cells and predispose to the development of a monoclonal plasma cell population.

### Role of the microenvironment

Studies have shown that the tumor microenvironment plays a pivotal role in lymphomagenesis and chemoresistance. Non-malignant cells such as stromal cells and macrophages are active components of the tumor that support survival and growth through direct and paracrine signaling. Likewise, tumor cells interact with non-malignant cells in a reciprocal fashion to promote tissue remodeling of the extracellular matrix and angiogenesis [60].

Due to the complexity of distinguishing a CHIP clone and a hematologic malignancy co-occurring in a single patient, the role of CH in the microenvironment has not been well characterized. However, one study of classical Hodgkin lymphoma (cHL) identified CHIP present in the reactive tissue cells of 2 patients (2/40). This reactive tissue is enriched in T-cells and often forms a supportive microenvironment for the Hodgkin cells via local suppression of anti-tumor responses and the production of soluble and surface-bound pro-survival factors. In these 2 cases, the CHIP clone ultimately comprised 60% and 94% of all reactive tissue cells. Interestingly, the CHIP clone in the latter case occurred completely independent of the Hodgkin clone, with unique mutations [61]. Despite the apparent domination of the microenvironment by a CHIP clone in these cases, it remains unclear how the clone contributes to cHL pathogenesis and whether removal of the clone would induce disease remission.

CHIP clones have also been documented to co-occur in patients with Waldenström macroglobulinemia (WM). This disease is characterized by the infiltration of lymphoplasmacytic B-cells into the bone marrow compartment and the development of IgM monoclonal gammopathy. In one patient sub-group, the presence of a CHIP clone was found in 14% of individuals and significantly increased the risk of progression from asymptomatic to symptomatic WM [62]. However, here too it is unclear how CH mediates this progression or whether the co-occurrence of CHIP and WM progression is merely reflective of other predisposing factors.

## Potential clinical implications of L-CH

### Autoimmunity

Due to their somatically rearranged lymphoid receptors, B and T-cell populations possess an unparalleled clonal diversity that permits the recognition of any pathogen via the activation of pathogen-specific lymphocytes. This process is tightly regulated to limit the activation and expansion of lymphocytes bearing self-reactive antigen receptors. However, just as mutations in L-CHIP subvert proliferation checkpoints and drive lymphomagenesis, so too can they subvert tolerance checkpoints and drive autoimmunity.

In the context of B-cells, a prime example of this phenomenon comes from an analysis of patient-derived B-cells producing rheumatoid factor. This study used single-cell sequencing to identify a “rogue” B-cell clone in the blood of 4 patients with type II cryoglobulinemic vasculitis. For two of these patients, somatic mutations in lymphoma driver genes only appear in a subset of the clone, clearly indicating these mutations were acquired in the mature peripheral cells. However, for the remaining two patients, mutations in lymphoma driver genes were present in all rheumatoid factor clones, raising the possibility that they derive centrally from a bone marrow progenitor. Notably, one patient possessed mutations in *TNFAIP3*, *KLHL6*, *ID3*, and *CCND3*, which have been previously linked to both HSC expansion and B cell activation [63–67].

In the context of T-cells, somatic loss of tolerance has previously been observed in seronegative autoimmune rheumatoid (sRA) arthritis, aplastic anemia (AA), and multiple sclerosis (MS). In sRA, patients are predisposed by specific HLA-I loci and frequently present clonal expansions of CD8 + T-cells. Notably, these clonal expansions contain somatic mutations found in the polyclonal T-cell repertoire, supporting central CH origination. In one studied patient, mutations in multiple cell cycle and activation pathways were found in the polyclonal CD8 + but not CD4 + population [68]. This observation suggests that the CHIP mutations alone may be insufficient to drive clonal expansion but can augment T-cell activation, as in the context of HLA-I risk alleles. Similar studies in AA identified CHIP in both CD8 and CD4 T-cell populations, although the candidate mutations were restricted to either the CD8 or CD4 lineage in most patient samples, indicating that AA is biased towards peripheral CH events [69]. Unfortunately, AA exerts a strong selective force on the bone marrow compartment and favors the expansion of central CH, which is a confounding factor when retrospectively inferring the contribution of central CH to AA development [70]. These findings are also echoed by a study of CD8 T-cells in multiple sclerosis (MS) patients, which identified clonal mutations of the *STAT3*, *SOCS1*, and *DNMT3A* genes. However, the low VAF of these mutations undermines any conclusive statements regarding CH in MS pathogenesis [71].

Some CHIP mutations affect both the T and B-cell compartments, as in the case of *FAS* deficiency leading to autoimmune lymphoproliferative syndrome (ALPS). These patients present with benign lymphoproliferation, hypergammaglobulinemia, an expansion of CD4/8 double-negative T-cells, and frequent autoimmune manifestations. Though ALPS is typically diagnosed during early childhood, a late-onset variant of the disease, ALPS-sFAS has been identified and linked to the clonal expansion of lymphocytes bearing *FAS* mutations. In this patient subgroup, targeted sequencing identified the somatic *FAS* variant concomitantly in T, B, and myeloid lineages, implicating central CH in disease pathogenesis [72–74].

Although somatic mutations have been recurrently found in the pathogenic lymphocytes of autoimmune disease, only a fraction of studies have performed the multi-lineage or single-cell analyses necessary to determine whether these mutations arise from central or peripheral CH. Regarding the contribution of central and peripheral L-CHIP to autoimmunity, we speculate that central L-CHIP bears a greater risk for the development of late-onset autoimmunity based on the need for L-CHIP mutations to coincide with cells expressing auto-reactive BCRs. This circumstance is likely to be met in a polyclonal population stemming from a mutated HSC, but not in a mutated mature cell with a single reactivity.

### Immunodeficiency

Like the age-related increase in autoimmune disease, there is also an age-related decline in protection from infection due to immune senescence. While immune senescence is an inevitable outcome in all individuals, CH can exacerbate this condition by allowing the expansion of less fit clones, essentially accelerating the onset of immune deficiency. Because CH is known to cause varying cytopenia’s via biased immune cell production and that immune deficiency is an expected outcome of cytopenia, we will focus our discussion here on the loss of immune cell function rather than the loss of immune cells.

The somatic loss of immune cell function in CH is most evident for mutations affecting cellular differentiation and is particularly well characterized for *TET2* loss of function mutations. Although *TET2* mutations are commonly associated with M-CHIP, it has now been shown in a mouse *Tet2* knock-out model that B and T-cell populations also expand proportionally and replace *Tet2* sufficient cells [75]. These lymphoid expansions are also reflected in the significant fraction of diffuse large B-cell, peripheral T-cell, and angioimmunoblastic T-cell lymphomas carrying *TET2* mutations, some of which have been traced back to an HSC mutation origin [76–79]. Notably, the loss of *Tet2* in B and T-cell populations produces cell intrinsic defects related to immune function. In a B-cell specific *Tet2* knock-out mouse model, epigenetic profiling found that loss of *Tet2* disrupted the expression of key regulators of plasma cell differentiation, such as *Prdm1*, via loss of cytosine hydroxymethylation at enhancer regions [77]. Similarly, loss of *TET2* also inhibits effector differentiation and cytokine production in T-cell populations via the same epigenetic mechanisms [80, 81]. These observations made in various *Tet2* mouse models are mirrored in patients bearing heterozygous or homozygous germline *TET2* mutations. While germline mutations in *TET2* do not permit the dissection of lymphocyte intrinsic effects, it is notable that these patients present similar disruptions in B and T-cell subtype frequencies and susceptibility to bacterial and viral infections [82, 83].

The pathogenic mechanism of somatic *TET2* mutation occurring centrally in the HSC is atypical in that it both supports clonal expansion and a reduced immune response. This is compared against other L-CHIP mutations which favor clonal expansion and cellular hyper-responsiveness as in the case in of autoimmunity. Despite this restriction, we speculate that any L-CHIP candidate gene could drive late-onset immunodeficiency if it could also cause primary immune deficiency when present as a heterozygous germline mutation. Therefore, this list may include mutations in genes such as *KMT2D* mutated in Kabuki syndrome, *PIK3CD* mutated in PI3 kinase delta syndrome (APDS), and *CARD11* mutated in B cell expansion with NF-κB and T-cell anergy (BENTA) [84–88].

### Solid tumors

Multiple studies have shown that CH is more common in patients with solid tumors. In an analysis of 31,717 cancer cases and 26,136 cancer-free controls, CH due to mCAs was found in 0.74% cancer-free individuals compared to 0.97% of cancer cases (OR = 1.25, p = 0.016), an association that was strengthened for cancer cases that had not undergone treatment (OR = 1.45, p = 0.0005) [13]. In an analysis of 8,810 paired blood and tumor samples, 25% percent of patients were found to have CHIP [4]. The higher incidence of CHIP in patients with solid tumors who have not undergone treatment and the previously discussed risk of immunodeficiency suggests that CHIP may predispose to the development of solid tumors by altering immune surveillance. CHIP has also been shown to decrease the survival of cancer patients in part due to an increased rate of primary cancer progression, providing further evidence that CHIP-related immune dysregulation may play an important role in the pathogenesis of cancers [4]. However, the co-occurrence of CHIP and solid tumors may also derive from inherited risk factors that globally increase genomic instability. Future studies will have to control for the degree of genomic instability to comprehensively evaluate the impact of CHIP on solid tumor development.

The response of the CHIP clone is both dependent on the mutation and the therapeutic regimen. A longitudinal study of solid-tumor cancer patients found that pre-existing CHIP clones with *TP53*, *PPM1D*, or *CHEK2* mutations significantly expanded following cytotoxic or radiation therapy, while mutations in other common CHIP genes such as *TET2* and *DNMT3A* remained stable or even decreased during therapy. Further, these trends increased with increasing chemotherapy exposure and were linked to specific classes of cytotoxic therapy, namely topoisomerase II inhibitors and carboplatin [89]. This supports the notion that L-CHIP increases the risk of therapy-related lymphoid malignancy.

### Donors of patients receiving HSCT

CHIP can arise in HSC transplant (HSCT) recipients by three distinct mechanisms: de novo mutations, a pre-existing clone in the donor HSCs, or a pre-existing clone in the recipient HSCs that survived bone marrow ablation. Cytotoxic therapy and engraftment following bone marrow ablation create a unique set of selective pressures that favor the more robust traits of a CHIP clone. The presence of CHIP among autologous-HSCT lymphoma and MM patients has been associated with multiple adverse outcomes including therapy related myeloid neoplasms (t-MNs) [90, 91]. One study found an increased 10-year incidence of t-MNs (25.3% vs. 9.9%, p < 0.01) and decreased 10-year survival in patients with CHIP (30.4% vs. 60.9%, p < 0.01) [92]. Based on these findings, there were concerns about the eligibility of donors with known carriers of CHIP mutations. Fick et al. investigated this question and found no difference in overall survival when comparing patients who received HSCs from a donor with CHIP mutations to patients who received HSCs from a donor without CHIP mutations. Notably, a subgroup analysis identified a survival benefit for patients with a non-complete response when the donor harbored CHIP mutations although the risk of chronic graft versus host disease was also higher (58.5% vs. 36.6%, p = 0.006) [93]. Further work has shown that engraftment of donor clones can alter inflammatory cytokine signaling leading to increased immunoreactivity towards both the primary cancer and host cells [94].

### CAR-T cell Therapy

Contrary to the detrimental consequences of L-CHIP enumerated above, mutations in *TET2*, *DNMT3A*, and potentially other CHIP mutations hold potential for engineering better CAR T-cells. A major challenge in CAR T-cell therapy is achieving expansion and persistence of the therapeutic cells in the recipient to maintain durable remission. In a serendipitous finding, Fraietta et al. identified a CAR T-cell recipient in which a single CAR T-cell expanded to dominate 94% of the CAR T-cell repertoire during the response peak and was still present over 4 years after infusion. This single CAR T-cell happened to have only a single hypomorphic *TET2* allele, as the lentivirus carrying the CAR transgene disrupted the other *TET2* allele upon gene insertion [95]. Surprisingly, although *Tet2* mutations alter T-cell differentiation and cytokine production, they do not appear to reduce cytotoxic activity, as observed in mouse models of lymphocytic choriomeningitis or *Listeria monocytogenes* infection. Further, *Tet2* knock-out T-cells are observed to preferentially acquire a central memory phenotype, which may contribute to the persistence of *TET2* hypomorphic CAR T-cells and maintenance of cancer remission [81]. These findings have since been reinforced by the discovery of superior proliferative and anti-tumor potential of *DNMT3A* knock-out CAR T cells. In a series of exhaustive in vitro and in vivo preclinical models, Prinzing et al. demonstrated that *DNMT3A* knock-out CAR T cells were resistant to exhaustion mediated by epigenetic silencing and maintained their therapeutic activity despite chronic antigen exposure [96].

## Conclusions

L-CH is a common condition among elderly adults that predisposes to a variety of lymphoid malignancies. The existence of these mutant clones years or even decades prior to overt malignancy provides a vital opportunity to identify high-risk patients and provide personalized monitoring and preventative care based on the size of the clone and the mutations it bears. Moreover, the ability to distinguish central and peripheral L-CH with single-cell techniques provides an important metric for stratifying patient risk, especially in the case of autoimmunity. Given these clones already possess a survival advantage, there remain concerns around the “fitness” of donors with L-CH for HSCT and the selection of chemotherapies for L-CH patients with non-hematological malignancy.

Continued research is necessary to further define the risks of L-CH and determine when or if intervention can benefit L-CH patients. The increasing awareness of this condition in the medical community is driving new ideas surrounding its management and prevention. As high throughput sequencing technologies continue to make their way into the clinic, new links will undoubtedly be established between L-CH and human disease.

## Data Availability

N/A
